# Mechanical model of steady-state and inflammatory conditions in patients with relapsing polychondritis: A review

**DOI:** 10.1097/MD.0000000000028852

**Published:** 2022-02-25

**Authors:** Jun Shimizu, Noboru Suzuki

**Affiliations:** Department of Immunology and Medicine, St. Marianna University School of Medicine, Kawasaki, Japan.

**Keywords:** interleukin-10, matrix metalloproteinase-3, regulatory T cells, relapsing polychondritis, short-chain fatty acids

## Abstract

Relapsing polychondritis (RP) is a multisystem inflammatory disorder, considered to associate with immune aberration.

Increased T helper type-1 cell-related cytokines were reported in RP patients. mRNA expressions of a regulatory T cell cytokine interleukin (IL)-10 increased, whereas pro-inflammatory cytokines IL1β and IL6 mRNA expressions decreased in freshly isolated peripheral blood mononuclear cells of RP patients compared with those in healthy individuals. Upon in vitro stimulation with mitogen, IL10 mRNA expressions decreased, and IL1β and IL6 mRNA expressions increased in RP patients.

This short-time dynamic change of gene expressions from anti-inflammatory to pro-inflammatory features of immune cells may be associated with the “relapsing” disease course of patients with RP. IL1β mRNA expressions of peripheral blood mononuclear cells exhibited positive correlations with serum matrix metalloproteinase (MMP)-3 concentrations in patients with respiratory involvement. Such positive correlation was not found in those without respiratory involvement.

In a metagenomic analysis, an altered composition of gut microbes was found, suggesting that microbe metabolites such as short-chain fatty acids may affect T cell responses of the patients.

In this review, the relationships among RP-related inflammatory molecules were summarized. The data support a hypothesis that the immune conditions are different between steady-state and inflammation in RP patients.

## 1 Introduction

Relapsing polychondritis (RP) is a chronic multisystem disorder of unknown etiology. The disease is characterized by recurrent episodic inflammation of cartilaginous tissues, such as the ears, nose, larynx, and tracheobronchial tree. Inflammation is often observed in proteoglycan-rich structures, such as the eyes, inner ears, heart, blood vessels, and kidneys.^[1]^

Recently, ear involvement and respiratory involvement of the patients were found to be mutually exclusive in the incidence at the last follow-up.^[2]^ Furthermore, based on the data, the patients were divided into 3 subgroups: patients with ear involvement, patients with respiratory involvement, and patients with both ear and respiratory involvement.^[3]^ Patients with ear involvement frequently demonstrate central nervous system (CNS) involvement, non-erosive arthritis, and conjunctivitis compared with other subgroups of patients.^[2,3]^ Patients with respiratory involvement frequently had saddle nose deformities and showed a progressive disease course.^[2,3]^ Patients with both involvement are frequently accompanied by cardiovascular involvement.^[2,3]^

Distinctive immunological characteristics were also observed in RP subgroups. Serum concentrations of matrix metalloproteinase (MMP)-3, the expression of which was recognized in RP lesions, increased significantly in RP patients with respiratory involvement compared with those without respiratory involvement.^[4]^ An adult-onset auto-inflammatory disease with somatic mutations of the ubiquitin activating E1 enzyme showed cell-intrinsic severe myeloid inflammation and developed auricular chondritis but not bronchial or laryngeal chondritis.^[5]^

Culture-based assays using peripheral blood mononuclear cells (PBMCs) were used to assess further immunological conditions in patients with RP. Interleukin (IL)-10 mRNA expression in freshly isolated PBMCs in RP patients was significantly higher than that in healthy individuals.^[6]^ IL10 is a major regulatory T (Treg)-related anti-inflammatory cytokine.^[7]^ In contrast to the results of freshly isolated PBMCs, in 6-hour-cultured PBMCs with mitogen stimulation, IL10 mRNA expression decreased significantly in RP patients compared with that in healthy individuals.^[6]^

24 hours after the initiation of the cell culture, RP PBMCs produced significantly increased mRNA levels of the inflammatory cytokine IL1β compared with those of healthy individuals.^[6]^ The short-term dynamics of PBMC gene expression from anti-inflammatory to pro-inflammatory features in the culture assay may be associated with relapsing and remitting clinical courses of patients with RP. Interestingly, IL1β mRNA expression correlated positively with serum concentrations of MMP3 in RP patients with respiratory involvement, but not in patients without respiratory involvement, suggesting that the molecular mechanisms of chondritis are different between patients with and without respiratory involvement.^[4]^

Simultaneously, remarkably altered gut microbiota composition were observed in patients with RP and the RP intestinal microenvironment was suggested to be related to skewed T cell function through the microbe metabolites.^[6]^

The aim of this review is to decipher the complex relationships among RP-related inflammatory molecules using our data and a literature review. A mechanical model of 2 disease conditions, namely steady-state and inflammatory conditions, was then proposed in the pathogenesis of RP.

## 2 Methods

### 2.1 Ethical statements

No ethical approval was required because this was a literature-based study. Our original clinical studies were approved by the institutional review boards of St. Marianna University School of Medicine (approval number 2406) and were registered with the University Hospital Medical Information Network-Clinical Trials Registry (UMIN000018937). This review was evaluated using the Scale for the Assessment of Narrative Review Articles (SANRA).

### 2.2 Searching methods

A literature review of the subgroup classification of patients with RP was conducted. Next, another literature review of immunology studies was conducted with a focus on RP-related inflammatory molecules. Relevant original English articles and case reports of RP were retrieved from the PubMed and MEDLINE databases in this review.

#### 2.2.1 Subgroup classification studies

A search for the subgroup classification of RP was conducted using the databases with the terms “relapsing polychondritis” and “classification.”

#### 2.2.2 Immunological studies

First, a search for papers was performed with RP-related inflammatory molecules, that is, “MMP3,” “inflammatory cytokines,” “inflammatory chemokines,” “T cell cytokines,” and “gut microbe metabolites,” adding to the term “relapsing polychondritis.” Second, an assessment of the relationships among “rheumatoid arthritis (RA),” “MMP3,” and “synovitis” was conducted using papers from the databases to compare the RA histopathological data of MMP3 with those of RP. Third, another search for articles was performed using the terms “RA,” “MMP3,” “T cells,” and “macrophages” for the assessment of the pathological finding in the immunocompetent cell migration and MMP3 expression to compare the RA data with those of RP.

## 3 Discussions/observations

### 3.1 Subtype classification studies in RP

With the terms “relapsing polychondritis” and “classification,” 34 papers were collected from the databases.

**Figure 1:**
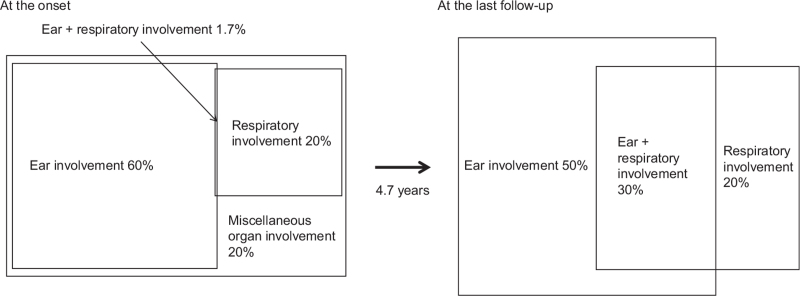
A subgroup analysis of RP patients at the disease onset (left panel) and the last follow-up (right panel). The subgroup analysis was conducted based on the incidence of auricular and respiratory involvement according to the findings of our previous study.^[2,3]^ At the onset of RP, patients were divided into 4 subgroups: patients with ear involvement (approximately 60%), patients with respiratory involvement (20%), patients with both ear and respiratory involvement (4 patients, 1.7%), and patients with miscellaneous organ involvement (20%), such as eye involvement, nasal involvement, nonerosive arthritis, inner ear involvement, skin involvement, and CNS involvement.^[10]^ After a mean follow-up of 4.7 years, the patients were divided into three subgroups: patients with ear involvement (approximately 50%), patients with respiratory involvement (20%), and patients with both ear and respiratory involvement (30%).^[3]^

A subgroup analysis of 239 patients with RP was performed using data from a multi-institutional study survey conducted in 2009.^[3]^ In the analysis, the patients were divided into 3 subgroups: patients with ear involvement (approximately 50% of 239 patients), patients with respiratory involvement (20%), and patients with both ear and respiratory involvement (30%) at the last follow-up (Fig. 1).^[3]^ All patients had ear involvement and/or respiratory involvement.^[3]^ Each subgroup demonstrated characteristic clinical phenotypes with several differences in the demographic data.^[3]^ Conjunctivitis, CNS involvement, and nonerosive arthritis are frequently observed in patients with ear involvement.^[3]^ Patients with respiratory involvement frequently have a saddle nose deformity with a progressive disease course.^[3]^ Patients with both involvement were characterized by cardiovascular involvement and a longer and progressive disease course.^[3]^ The data suggest that the subgroup analysis may play a role in the development of classification criteria for RP.

Subsequently, the same survey data were analyzed with a focus on patient symptoms at disease onset using similar methods.^[8]^ At the onset of RP, patients were divided into 4 subgroups: patients with ear involvement (approximately 60% of all analyzed patients), patients with respiratory involvement (20%), patients with both ear and respiratory involvement (4 patients, 1.7%), and patients with miscellaneous organ involvement (20%), such as eye involvement, nasal involvement, nonerosive arthritis, inner ear involvement, skin involvement, and CNS involvement (Fig. 1).^[8]^ One-third of patients with ear involvement at disease onset developed respiratory involvement later in the disease course with a relatively high mortality rate,^[8]^ suggesting the importance of prophylactic strategies for RP patients with ear involvement.

Consistent with our results, recent studies have identified several subgroups utilizing patients’ demographic and clinical information.^[9–11]^ For example, a clinical study of RP identified 3 subgroups based on demographic data (2 variables), clinical manifestations (14 variables), and use of immunosuppressants and biologic agents.^[9]^ In this study, RP patients in subgroup type 1 were associated with myelodysplastic syndrome, cutaneous involvement, and cardiovascular involvement without respiratory involvement. RP patients in subgroup type 2 were relatively young and had frequent tracheobronchial involvement and/or laryngeal involvement. Subgroup type 3 patients demonstrated a mild disease course and low numbers of clinical manifestations and treatments. Another subgroup analysis divided the patients into 3 subgroups based on clinical manifestations (8 variables).^[10]^ Patients in subgroup type 1 were defined as having extensive cartilage damage and ear involvement. Patients in subgroup type 2 had lower airway involvement and lower incidence of nasal involvement and ear involvement. Patients in subgroup type 3 were characterized by tenosynovitis/synovitis and ear involvement with a lower incidence of respiratory involvement and nasal involvement. Certainly, the pathogenesis of RP seems to be influenced by affected organs, at least in some patients.

Recently, a single-center physician global assessment was conducted to assess disease activity and progression in patients with RP.^[12]^ The authors concluded that most patients with RP demonstrated persistent disease activity rather than relapsing and remitting the clinical course, regardless of their treatment. The disease processes of RP may be more complicated than previously thought, and clinical investigations should be performed with more frequent monitoring.

### 3.2 Immunological studies in RP

In the first search of immunological studies, 2 papers on MMP3, 19 papers on inflammatory cytokines, 3 papers on inflammatory chemokines, 16 papers on T cell cytokines, and 2 papers on gut microbe metabolites were retrieved in patients with RP. In the second search, 71 papers on MMP3 and synovitis were collected in patients with RA. In the third search, 43 papers on MMP3, T cells, and macrophages were collected in patients with RA. The literature review data with our research findings were summarized in this review.

#### 3.2.1 MMP3

MMP3 is an extracellular proteolytic enzyme that cleaves many substrates.^[13]^ Serum levels of MMP3 increased in patients with rheumatic diseases, including RA, psoriatic arthritis, and polymyalgia rheumatica.^[14]^ MMP3 expressions in pannus tissues was significantly higher in RA patients than in patients with osteoarthritis, and IL1β promoted MMP3 production in fibroblasts.^[15]^ Synovectomy decreased serum levels of MMP3.^[16]^ Serum MMP3 levels in patients with RA correlated positively with MMP3 levels in the supernatants of synovial tissue homogenates.^[17]^ In the biopsy specimens of patients with RA, the number of CD3+ T cells and CD68+ macrophages increased in the synovial tissue^[18]^ and the adjacent cartilage-pannus junction.^[19]^ Infiltrating CD4+ T cell counts in the synovial membrane correlated with synovial fluid MMP3 concentrations.^[20]^ The numbers of inflammatory cells decreased in surgical samples of patients with advanced RA.^[18]^

Serum MMP3 levels increased significantly in patients with RP compared to those in healthy individuals.^[3,21]^ In the early stages of the disease, immunocompetent cells, such as macrophages, lymphocytes, neutrophils, and plasma cells, infiltrate into the perichondrium^[22]^ through the blood vessels.^[23,24]^ A histopathological examination of auricular chondritis in patients with RP demonstrated that MMP3 expression was observed not only in the degenerating chondrocytes but also in the perichondrium tissues with relatively weak staining.^[25]^ The MMP3 positive cell numbers showed a positive correlation with the number of apoptotic cells. CD4+ helper T cells and CD68+ macrophages migrated mainly into the perichondrium of patients with RP.^[25]^

These data suggest that chondritis of RP patients and synovitis of RA patients may share common features in the underlying cellular and molecular mechanisms to some extent, especially in view of the roles of MMP3 in inflammation, at least at the early stages of the disease in some patients.

#### 3.2.2 Inflammatory cytokines and chemokines

Several articles have described the overexpression of inflammatory cytokines and chemokines in patients with RP. Enzyme-linked immunosorbent assay and bead-based assays demonstrated elevated levels of IL8, CCL2,^[26]^ and CCL4^[21,26]^ in patients with RP compared with healthy individuals. Soluble forms of triggering receptor expressed on myeloid cells (TREM)-1 increased significantly in the sera of patients with active RP compared with those with inactive RP.^[21]^ From these results, it is conceivable that monocytes/macrophages and neutrophils are over-activated in the peripheral blood of patients with RP.^[26]^

Previously, the levels of inflammatory cytokine mRNA expression in PBMCs of patients with RP were measured and the data were compared with those of healthy individuals using an ex vivo culture assay with mitogen stimulation.^[6]^ The expression of IL1β, IL6, and tumor necrosis factor (TNF)-α decreased significantly in freshly isolated PBMCs of RP patients compared with that in healthy individuals.^[6]^ IL1β expression increased significantly and IL6 increased moderately in 24-hour-cultured RP PBMCs compared with that in healthy individuals.^[6]^ PBMC TNFα mRNA expression in RP patients remained at significantly low levels throughout the 24-hour-culture period.^[6]^

Simultaneously, serum MMP3 levels increased significantly in RP patients with respiratory involvement compared with those in RP patients without respiratory involvement.^[4]^ Serum MMP3 concentrations showed positive correlations with IL1β and IL6 mRNA expressions in 24-hour-cultured PBMCs in RP patients with respiratory involvement, but not in RP patients without respiratory involvement.^[4]^ These analyses suggest that IL1β and IL6 may be more effective in inducing MMP3 in laryngotracheal cartilage than those in cartilaginous tissues of other organs,^[27]^ presumably through the overexpression of several RP-associated autoantigens, including type II collagen^[28]^ and matrilin1.^[29]^

Skewed T cell responses may be associated with the differences in mRNA expression between IL1β/IL6 and TNFα, as mentioned in the next section.

#### 3.2.3 T cell cytokines

In T cell-related cytokines, interferon (IFN)γ,^[21,30]^ IL10,^[26]^ and IL12^[30]^ increased in the sera of patients with RP. Recent flow cytometry analysis of the T cell subset indicated that Th2 cells and Treg cells decreased significantly in patients with RP compared with healthy individuals, and Th1 and Th17 cells were comparable between them.^[31]^ To date, it has been generally assumed that the Th1 response is enhanced in the peripheral blood of RP patients.^[19]^

In a study of RP PBMC gene expression,^[6]^ IL10 mRNA expression in freshly isolated PBMCs increased significantly in patients with RP compared with those in healthy individuals, consistent with previous data of high serum concentrations of IL10 in RP patients.^[26]^ In contrast, IL10 mRNA expressions in 6-hour- and 24-hour-cultured PBMCs decreased significantly in RP patients compared with that in healthy individuals. IL10 is a major Treg-related cytokine, and the Treg cell-derived IL10 is thought to be important in reducing the immune response against viruses and bacteria.^[7]^ IL10 deletion induced severe spontaneous inflammation in the intestine, probably because of intolerance to gut microbes.^[32]^ Thus, these data revealed that RP PBMCs demonstrated short-time dynamics in gene expression from anti-inflammatory to pro-inflammatory features.

Interestingly, the cartilage-protective effects of IL10 were observed in type II collagen-^[33]^ and matrilin1-induced^[34]^ arthritis models. In an in vitro experiment using mechanically injured cartilage, IL10 supplementation increased type II collagen expression^[35]^ and reduced MMP3 expression^[36]^ in tissues. Type II collagen mRNA was increased in primary chondrocytes upon supplementation with IL10.^[37]^

**Figure 2:**
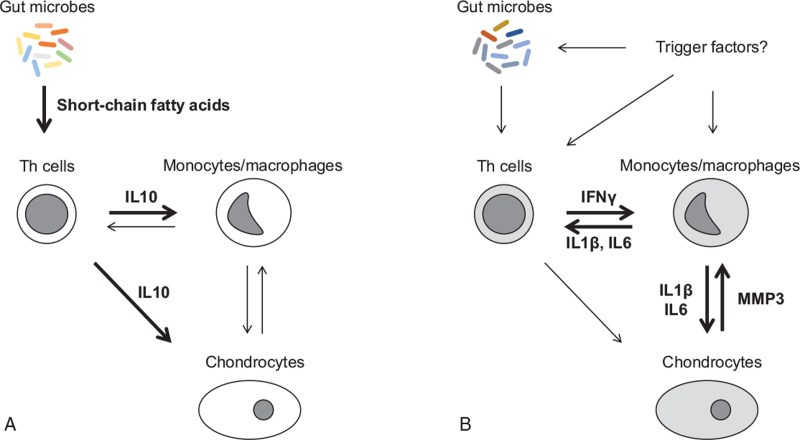
Schematic representation of pathogenic immune responses in (A) freshly isolated peripheral blood mononuclear cells (PBMCs) and (B) cultured PBMCs with mitogen stimulation in patients with RP. The data suggest that the changes of mRNA expressions in the PBMC experiments may mimic the transition of the 2 different immunological conditions of patients with RP, namely (A) steady-state condition and (B) inflammatory condition. (A) Propionate-producing gut microbes promote continuously IL10-producing Treg cell differentiation. The Treg cells may inhibit inflammatory cytokine production of monocytes/macrophages. Treg cell secreting IL10 has the potential to protect cartilaginous tissues from inflammation in patients with RP. (B) Trigger factors with inflammation may induce anergy and exhaustion in RP Treg cells. Reduced IL10 production associates with IFNγ production of effector T cells and IL1β and IL6 release from monocytes/macrophages, which promotes damage of chondrocytes. MMP3 secreted by the chondrocytes aggravates chondritis in an autocrine manner. These molecules may relate subsequently to severe chondritis in patients with RP.

These data suggest that the changes in mRNA expression in the ex vivo assay may mimic the transition of the 2 different immunological conditions of patients with RP, namely “steady-state condition” and “inflammatory condition” (Fig. 2).

In the steady-state condition of RP patients (Fig. 2A), similar to the experimental results of freshly isolated PBMCs, gut microbial metabolites, as described in the next section, may promote continuous IL10-producing Treg cell differentiation. Treg cells may inhibit inflammatory cytokine production by monocytes/macrophages.^[38]^ Treg cell secreting IL10 have the potential to protect cartilaginous tissues from inflammation in RP patients.^[33–37]^

In the inflammatory condition of RP patients (Fig. 2B), similar to the results of 6-hour- and 24-hour-cultured PBMCs with mitogen stimulation, trigger factors with inflammation may induce anergy and exhaustion in Treg cells. Reduced IL10 production is associated with IFNγ production by effector T cells^[21]^ and IL1β and IL6 release from monocytes/macrophages,^[38]^ which promotes damage to chondrocytes.^[39]^ MMP3 secreted by chondrocytes aggravates chondritis in an autocrine manner.^[25]^ These molecules may subsequently be related to severe chondritis in patients with RP.

Previously, T cell-related cytokines, that is, IFNγ,^[40]^ IL4,^[40]^ and IL10^[41,42]^ were reported to be associated with TNFα mRNA destabilization through modification of RNA binding proteins in macrophages. IL10-producing Treg cell dominance of PBMCs in RP patients may reduce TNFα mRNA expression of monocytes/macrophages in the steady-state immune condition of patients with RP (Fig. 2A). Under inflammatory conditions (Fig. 2B), IFNγ may induce the mRNA destabilization of monocytes/macrophages in RP patients. Further studies are warranted to assess the differences in the inflammatory cytokine responses of RP PBMCs against the stimulation of anti-inflammatory compounds, such as disease-modifying anti-rheumatic drugs and biologic agents.

#### 3.2.4 Gut microbe metabolites

Inside the intestinal tract, especially in the large intestine, many bacteria reside mainly in the mucus and are closely related to host immunity through their and host metabolites.^[43]^ Gut microbes are known to promote immune system maturation through metabolites, and germ-free mice demonstrate low induction of secretory IgA and CD4+/CD8+ T cells in the intestine.^[44]^ A recent study revealed that human fetal tissues in gestational week 12-22 contained cultivable bacteria and the genomes in several organs, such as the gut, lung, and spleen, and the fetal T cells reacted with the bacterial antigens.^[45]^ The researchers assumed that the immune competency and priming of fetuses were established from the early stages of gestation using information from the placenta. Microbes may have a close relationship with host immune function during development.

The metabolites include short-chain fatty acids (SCFAs), bile acids, choline metabolites, phenol, benzoyl, phenyl derivatives, and indole derivatives.^[46]^ In the metabolites, SCFAs are the most important gut microbe-derived molecules and exhibit various effects on commensal and host homeostasis.^[46]^

SCFAs consist mainly of acetate, propionate, and butyrate, and are produced by the fermentation of microbes from non-digestible carbohydrates.^[47]^ The Fermentation to acetate is performed by relatively large numbers of intestinal bacteria, but a few gut microbes produce propionate and butyrate.^[48]^ SCFAs are utilized as energy sources for the body, and their consumption is observed with SCFA-specific distribution.^[47]^ A large proportion of butyrate is consumed in the colon epithelium cells and maintains the integrity of the mucosal barrier by upregulating the tight junction.^[49,50]^ Propionate involves in hepatic gluconeogenesis and acts as a modulator of glucose homeostasis.^[51,52]^ Acetate is consumed in the periphery, such as the brain, muscle, pancreas, and adipose tissue.^[53,54]^

Recently, it was shown that SCFAs increased IL10-expressing Treg cells in the intestine and reduced inflammation in mouse disease models.^[55–58]^ SCFAs controlled T cell differentiation through epigenetic modification.^[55,57,58]^ Based on the data, a metagenomic analysis was conducted to assess alterations in gut microbial composition and gene function in patients with RP.^[6]^

As expected, propionate-producing bacteria increased significantly in the microbe proportion of RP patients compared with that of healthy individuals.^[6]^ RP prevalent gene functions of the metagenomic data associated with the succinate-propionate pathway, suggesting excess propionate production in the intestine of RP patients.^[6]^ These data may support the hypothesis that gut microbes provide substantial Treg cell-inducing stimulation to the intestinal immune system through the production of propionate (Fig. 2A).

A large portion of intestinal Treg cells is thought to originate from thymus-derived Treg cells after homing^[59]^ during the early stages of life,^[60]^ suggesting that the cells recognize self and microbial antigens.^[59,61,62]^ Indeed, human type II collagen demonstrated 32% to 47% identity and 36% to 52% similarity in the protein sequences to the bacterial species.^[63]^ Our basic local alignment search tool protein database search identified 94 bacterial species with significant homology to human matrilin1 (23%–52% identity and 43%–69% similarity).

Furthermore, administration of SCFAs alone in germ-free mice did not induce Treg cells in the intestine, indicating an indispensable role of bacterial stimulation in Treg cell differentiation.^[57]^ These findings refocused on the molecular mimicry between the host and microbes from the viewpoint of recent metagenomic data.

## 4 Conclusions

Here, recent findings on the role of inflammatory molecules in patients with RP are summarized using our data and a literature review. The underlying molecular mechanisms of the patients were discussed using the 2 conditions in Figure 2, namely patients in steady-state condition and patients in inflammatory condition. The presented approach will have great potential in the development of therapeutic strategies based on clinical features more accurately in patients with RP.

## Author contributions

**Conceptualization:** Jun Shimizu.

**Writing – original draft:** Jun Shimizu.

**Writing – review & editing:** Jun Shimizu, Noboru Suzuki.

## References

[1] Letko E Zafirakis P Baltatzis S Voudouri A Livir-Rallatos C Foster CS. Relapsing polychondritis: a clinical review. Semin Arthritis Rheum 2002;31:384–95. 12077711 10.1053/sarh.2002.32586

[2] Shimizu J Yamano Y Yudoh K Suzuki N. Organ involvement pattern suggests subgroups within relapsing polychondritis: comment on the article by Dion et al. Arthritis Rheumatol 2018;70:148–9. 28941194 10.1002/art.40330

[3] Shimizu J Yamano Y Kawahata K Suzuki N. Relapsing polychondritis patients were divided into three subgroups: patients with respiratory involvement (R subgroup), patients with auricular involvement (A subgroup), and overlapping patients with both involvements (O subgroup), and each group had distinctive clinical characteristics. Medicine (Baltimore) 2018;97:e12837. 30334986 10.1097/MD.0000000000012837 PMC6211876

[4] Shimizu J Wakisaka S Suzuki T Suzuki N. Serum MMP3 correlated with IL1β mRNA expressions of PBMC in relapsing polychondritis patients with respiratory involvement. ACR Open Rheum 2021;3:636–41. 10.1002/acr2.11301 PMC8449037 34289257

[5] Beck DB Ferrada MA Sikora KA. Somatic mutations in UBA1 and severe adult-onset autoinflammatory disease. N Engl J Med 2020;383:2628–38. 33108101 10.1056/NEJMoa2026834 PMC7847551

[6] Shimizu J Kubota T Takada E. Propionate-producing bacteria in the intestine may associate with skewed responses of IL10-producing regulatory T cells in patients with relapsing polychondritis. PLoS One 2018;13:e0203657. 30235279 10.1371/journal.pone.0203657 PMC6147427

[7] Neumann C Scheffold A Rutz S. Functions and regulation of T cell-derived interleukin-10. Semin Immunol 2019;44:101344. 31727465 10.1016/j.smim.2019.101344

[8] Shimizu J Yamano Y Kawahata K Suzuki N. Elucidation of predictors of disease progression in patients with relapsing polychondritis at the onset: potential impact on patient monitoring. BMC Rheumatol 2020;4:41. 32944685 10.1186/s41927-020-00141-8 PMC7488391

[9] Dion J Costedoat-Chalumeau N Sène D. Relapsing polychondritis can be characterized by three different clinical phenotypes: analysis of a recent series of 142 patients. Arthritis Rheumatol 2016;68:2992–3001. 27331771 10.1002/art.39790

[10] Ferrada M Rimland CA Quinn K. Defining clinical subgroups in relapsing polychondritis: a prospective observational cohort study. Arthritis Rheumatol 2020;72:1396–402. 32249511 10.1002/art.41270 PMC8672710

[11] Zhang L Yun S Wu T. Clinical patterns and the evolution of relapsing polychondritis based on organ involvement: a Chinese retrospective cohort study. Orphanet J Rare Dis 2021;16:225. 34001193 10.1186/s13023-021-01861-x PMC8130285

[12] Rose E Ferrada MA Quinn KA. Physician global assessment as a disease activity measure for relapsing polychondritis. Arthritis Care Res (Hoboken) 2021;doi: 10.1002/acr.24574. Online ahead of print. 10.1002/acr.24574 PMC8339175 33544969

[13] Cui N Hu M Khalil RA. Biochemical and biological attributes of matrix metalloproteinases. Prog Mol Biol Transl Sci 2017;147:01–73. 10.1016/bs.pmbts.2017.02.005 PMC5430303 28413025

[14] Ribbens C Martin y Porras M Franchimont N. Increased matrix metalloproteinase-3 serum levels in rheumatic diseases: relationship with synovitis and steroid treatment. Ann Rheum Dis 2002;61:161–6. 11796404 10.1136/ard.61.2.161 PMC1753989

[15] Ainola MM Mandelin JA Liljeström MP Li TF Hukkanen MV Konttinen YT. Pannus invasion and cartilage degradation in rheumatoid arthritis: involvement of MMP-3 and interleukin-1beta. Clin Exp Rheumatol 2005;23:644–50. 16173240

[16] Lipina M Makarov M Makarov S Novikov A. The degree of cartilage degradation assessed by serum biomarker levels changes after arthroscopic knee synovectomy in rheumatoid arthritis patients. Int Orthop 2017;41:2259–64. 28889180 10.1007/s00264-017-3634-8

[17] Kobayashi A Naito S Enomoto H. Serum levels of matrix metalloproteinase 3 (stromelysin 1) for monitoring synovitis in rheumatoid arthritis. Arch Pathol Lab Med 2007;131:563–70. 17425385 10.5858/2007-131-563-SLOMMS

[18] Smeets TJ Barg EC Kraan MC Smith MD Breedveld FC Tak PP. Analysis of the cell infiltrate and expression of proinflammatory cytokines and matrix metalloproteinases in arthroscopic synovial biopsies: comparison with synovial samples from patients with end stage, destructive rheumatoid arthritis. Ann Rheum Dis 2003;62:635–8. 12810425 10.1136/ard.62.7.635 PMC1754593

[19] Smeets TJ Kraan MC Galjaard S Youssef PP Smith MD Tak PP. Analysis of the cell infiltrate and expression of matrix metalloproteinases and granzyme B in paired synovial biopsy specimens from the cartilage-pannus junction in patients with RA. Ann Rheum Dis 2001;60:561–5. 11350843 10.1136/ard.60.6.561 PMC1753677

[20] Fraser A Fearon U Billinghurst RC. Turnover of type II collagen and aggrecan in cartilage matrix at the onset of inflammatory arthritis in humans: relationship to mediators of systemic and local inflammation. Arthritis Rheumatol 2003;48:3085–95. 10.1002/art.11331 14613270

[21] Sato T Yamano Y Tomaru U. Serum level of soluble triggering receptor expressed on myeloid cells-1 as a biomarker of disease activity in relapsing polychondritis. Mod Rheumatol 2014;24:129–36. 24261769 10.3109/14397595.2013.852854

[22] Kumakiri K Sakamoto T Karahashi T Mineta H Takebayashi S. A case of relapsing polychondritis preceded by inner ear involvement. Auris Nasus Larynx 2005;32:71–6. 15882830 10.1016/j.anl.2004.09.012

[23] McAdam LP O’Hanlan MA Bluestone R Pearson CM. Relapsing polychondritis: prospective study of 23 patients and a review of the literature. Medicine (Baltimore) 1976;55:193–215. 775252

[24] Shaul SR Schumacher HR. Relapsing polychondritis. Electron microscopic study of ear cartilage. Arthritis Rheumatol 1975;18:617–25. 10.1002/art.1780180614 128363

[25] Ouchi N Uzuki M Kamataki A Miura Y Sawai T. Cartilage destruction is partly induced by the internal proteolytic enzymes and apoptotic phenomenon of chondrocytes in relapsing polychondritis. J Rheumatol 2011;38:730–7. 21239745 10.3899/jrheum.101044

[26] Stabler T Piette JC Chevalier X Marini-Portugal A Kraus VB. Serum cytokine profiles in relapsing polychondritis suggest monocyte/macrophage activation. Arthritis Rheumatol 2004;50:3663–7. 10.1002/art.20613 15529362

[27] Arnaud L Mathian A Haroche J Gorochov G Amoura Z. Pathogenesis of relapsing polychondritis: a 2013 update. Autoimmun Rev 2014;13:90–5. 24051104 10.1016/j.autrev.2013.07.005

[28] Foidart JM Abe S Martin GR. Antibodies to type II collagen in relapsing polychondritis. N Engl J Med 1978;299:1203–7. 714080 10.1056/NEJM197811302992202

[29] Hansson AS Heinegård D Piette JC Burkhardt H Holmdahl R. The occurrence of autoantibodies to matrilin 1 reflects a tissue-specific response to cartilage of the respiratory tract in patients with relapsing polychondritis. Arthritis Rheumatol 2001;44:2402–12. 10.1002/1529-0131(200110)44:10<2402::aid-art405>3.0.co;2-l 11665983

[30] Kraus VB Stabler T Le ET Saltarelli M Allen NB. Urinary type II collagen neoepitope as an outcome measure for relapsing polychondritis. Arthritis Rheum 2003;48:2942–8. 14558101 10.1002/art.11281

[31] Hu FY Wang J Zhang SX. Absolute reduction of peripheral regulatory T cell in patients with relapsing polychondritis. Clin Exp Rheumatol 2021;39:487–93. 32573423

[32] Kühn R Löhler J Rennick D Rajewsky K Müller W. Interleukin-10-deficient mice develop chronic enterocolitis. Cell 1993;75:263–74. 8402911 10.1016/0092-8674(93)80068-p

[33] Tian S Yan Y Qi X Li X Li Z. Treatment of type II collagen-induced rat rheumatoid arthritis model by interleukin 10 (IL10)-mesenchymal stem cells (BMSCs). Med Sci Monit 2019;25:2923–34. 31005957 10.12659/MSM.911184 PMC6489530

[34] Hansson AS Johannesson M Svensson L Nandakumar KS Heinegård D Holmdahl R. Relapsing polychondritis, induced in mice with matrilin 1, is an antibody- and complement-dependent disease. Am J Pathol 2004;164:959–66. 14982849 10.1016/S0002-9440(10)63183-5 PMC1614711

[35] Behrendt P Feldheim M Preusse-Prange A. Chondrogenic potential of IL-10 in mechanically injured cartilage and cellularized collagen ACI grafts. Osteoarthritis Cartilage 2018;26:264–75. 29169959 10.1016/j.joca.2017.11.007

[36] Behrendt P Preusse-Prange A Klüter T. IL-10 reduces apoptosis and extracellular matrix degradation after injurious compression of mature articular cartilage. Osteoarthritis Cartilage 2016;24:1981–8. 27349464 10.1016/j.joca.2016.06.016

[37] Jung YK Kim GW Park HR. Role of interleukin-10 in endochondral bone formation in mice: anabolic effect via the bone morphogenetic protein/Smad pathway. Arthritis Rheumatol 2013;65:3153–64. 10.1002/art.38181 24022823

[38] de Waal Malefyt R Abrams J Bennett B Figdor CG de Vries JE. Interleukin 10 (IL-10) inhibits cytokine synthesis by human monocytes: an autoregulatory role of IL-10 produced by monocytes. J Exp Med 1991;174:1209–20. 1940799 10.1084/jem.174.5.1209 PMC2119001

[39] Kapoor M Martel-Pelletier J Lajeunesse D Pelletier JP Fahmi H. Role of proinflammatory cytokines in the pathophysiology of osteoarthritis. Nat Rev Rheumatol 2011;7:33–42. 21119608 10.1038/nrrheum.2010.196

[40] Suk K Erickson KL. Differential regulation of tumour necrosis factor-alpha mRNA degradation in macrophages by interleukin-4 and interferon-gamma. Immunology 1996;87:551–8. 8675208 10.1046/j.1365-2567.1996.500561.x PMC1384132

[41] Rajasingh J Bord E Luedemann C. IL-10-induced TNF-alpha mRNA destabilization is mediated via IL-10 suppression of p38 MAP kinase activation and inhibition of HuR expression. FASEB J 2006;20:2112–4. 16935932 10.1096/fj.06-6084fje

[42] Inoue M Arikawa T Chen YH. T cells down-regulate macrophage TNF production by IRAK1-mediated IL-10 expression and control innate hyperinflammation. Proc Natl Acad Sci U S A 2014;111:5295–300. 24706909 10.1073/pnas.1321427111 PMC3986140

[43] Rooks MG Garrett WS. Gut microbiota, metabolites and host immunity. Nat Rev Immunol 2016;16:341–52. 27231050 10.1038/nri.2016.42 PMC5541232

[44] Round JL Mazmanian SK. The gut microbiota shapes intestinal immune responses during health and disease. Nat Rev Immunol 2009;9:313–23. 19343057 10.1038/nri2515 PMC4095778

[45] Mishra A Lai GC Yao LJ. Microbial exposure during early human development primes fetal immune cells. Cell 2021;184:3394–409. e20. 34077752 10.1016/j.cell.2021.04.039 PMC8240556

[46] Nicholson JK Holmes E Kinross J. Host-gut microbiota metabolic interactions. Science 2012;336:1262–7. 22674330 10.1126/science.1223813

[47] Morrison DJ Preston T. Formation of short chain fatty acids by the gut microbiota and their impact on human metabolism. Gut Microbes 2016;7:189–200. 26963409 10.1080/19490976.2015.1134082 PMC4939913

[48] Louis P Flint HJ. Formation of propionate and butyrate by the human colonic microbiota. Environ Microbiol 2017;19:29–41. 27928878 10.1111/1462-2920.13589

[49] Fachi JL Felipe JS Pral LP. Butyrate protects mice from Clostridium difficile-induced colitis through an HIF-1-dependent mechanism. Cell Rep 2019;27:750–61. e7. 30995474 10.1016/j.celrep.2019.03.054

[50] Kelly CJ Zheng L Campbell EL. Crosstalk between microbiota-derived short-chain fatty acids and intestinal epithelial HIF augments tissue barrier function. Cell Host Microbe 2015;17:662–71. 25865369 10.1016/j.chom.2015.03.005 PMC4433427

[51] Chambers ES Byrne CS Morrison DJ. Dietary supplementation with inulin-propionate ester or inulin improves insulin sensitivity in adults with overweight and obesity with distinct effects on the gut microbiota, plasma metabolome and systemic inflammatory responses: a randomized cross-over trial. Gut 2019;68:1430–8. 30971437 10.1136/gutjnl-2019-318424 PMC6691855

[52] den Besten G Lange K Havinga R. Gut-derived short-chain fatty acids are vividly assimilated into host carbohydrates and lipids. Am J Physiol Gastrointest Liver Physiol 2013;305:G900–10. 24136789 10.1152/ajpgi.00265.2013

[53] Bloemen JG Venema K van de Poll MC Olde Damink SW Buurman WA Dejong CH. Short chain fatty acids exchange across the gut and liver in humans measured at surgery. Clin Nutr 2009;28:657–61. 19523724 10.1016/j.clnu.2009.05.011

[54] van Eijk HM Bloemen JG Dejong CH. Application of liquid chromatography-mass spectrometry to measure short chain fatty acids in blood. J Chromatogr B Analyt Technol Biomed Life Sci 2009;877:719–24. 10.1016/j.jchromb.2009.01.039 19230798

[55] Smith PM Howitt MR Panikov N. The microbial metabolites, short-chain fatty acids, regulate colonic Treg cell homeostasis. Science 2013;341:569–73. 23828891 10.1126/science.1241165 PMC3807819

[56] Haghikia A JoÈrg S Duscha A. Dietary fatty acids directly impact central nervous system autoimmunity via the small intestine. Immunity 2015;43:817–29. 26488817 10.1016/j.immuni.2015.09.007

[57] Furusawa Y Obata Y Fukuda S. Commensal microbe-derived butyrate induces the differentiation of colonic regulatory T cells. Nature 2013;504:446–50. 24226770 10.1038/nature12721

[58] Park J Kim M Kang SG. Short-chain fatty acids induce both effector and regulatory T cells by suppression of histone deacetylases and regulation of the mTOR-S6K pathway. Mucosal Immunol 2015;8:80–93. 24917457 10.1038/mi.2014.44 PMC4263689

[59] Cebula A Seweryn M Rempala GA. Thymus-derived regulatory T cells contribute to tolerance to commensal microbiota. Nature 2013;497:258–62. 23624374 10.1038/nature12079 PMC3711137

[60] Korn LL Hubbeling HG Porrett PM Yang Q Barnett LG Laufer TM. Regulatory T cells occupy an isolated niche in the intestine that is antigen independent. Cell Rep 2014;9:1567–73. 25482559 10.1016/j.celrep.2014.11.006 PMC12079782

[61] Tanoue T Atarashi K Honda K. Development and maintenance of intestinal regulatory T cells. Nat Rev Immunol 2016;16:295–309. 27087661 10.1038/nri.2016.36

[62] Whibley N Tucci A Powrie F. Regulatory T cell adaptation in the intestine and skin. Nat Immunol 2019;20:386–96. 30890797 10.1038/s41590-019-0351-z

[63] Alam J Kim YC Choi Y. Potential role of bacterial infection in autoimmune diseases: a new aspect of molecular mimicry. Immune Netw 2014;14:07–13. 10.4110/in.2014.14.1.7 PMC3942510 24605075

